# Kinetic modeling of heterogeneous esterification reaction using initial reaction rate analysis: data extraction and evaluation of mass transfer criteria

**DOI:** 10.1016/j.dib.2020.106027

**Published:** 2020-07-15

**Authors:** Philippe M. Heynderickx, Somboon Chaemchuen, Francis Verpoort

**Affiliations:** a Center for Environmental and Energy Research (CEER) – Engineering of Materials via Catalysis and Characterization, Ghent University Global Campus, 119-5 Songdomunhwa-Ro, Yeonsu-Gu, Incheon, 406-840, South Korea; b Department of Green Chemistry and Technology (BW24), Faculty of Bioscience Engineering, Ghent University, 653 Coupure Links, Ghent, B-9000, Belgium; c Laboratory of Organometallics, Catalysis and Ordered Materials, State Key Laboratory of Advanced Technology for Materials Synthesis and Processing, Wuhan University of Technology, Wuhan, 430070, P.R. China; d National Research Tomsk Polytechnic University, Lenin Avenue 30, 634050, Tomsk, Russian Federation

**Keywords:** Intrinsic kinetic data, Initial reaction rate analysis, Model discrimination, Metal organic framework

## Abstract

This data article provides detailed guidance to obtain heterogeneous reaction rate expressions and the corresponding initial reaction rates and their application. Explanation is provided to deal with specific criteria to rule out internal and external concentration gradients, so that the usage of intrinsic catalytic data is guaranteed. Overall, the main goal is to provide an easy tool to evaluate both aforementioned results by simple plug-and-play of available reaction data.

## Abstract (author highlights)

Value of the Data

• The presented data and corresponding data treatment can be put forward by other researchers in order to guarantee the acquisition of intrinsic experimental data for catalytic reactions.
• The presented data can be used as an example to set up typical heterogeneous esterification reactions by researchers working on catalytic systems with the specific purpose of kinetic modeling.
• Data treatment in order to calculate initial reaction rates is explained in detail with example. This has a high applicability and very easy practicability for users in the research field of heterogeneous catalysis.
• Concentration gradients, which might destroy the intrinsic character of the experimental data, can be ruled out via simple criteria. How to use the data is explained in this manuscript.

## Nomenclature

**Roman symbols**
a: coefficient in Eq. (1) (mol mol^−1^)
a_v_: area to volume ratio (m^2^ m^−3^)
b: coefficient in Eq. (1); coefficient in Eq. (27) (s^−1^, h^−1^; dep.)
C: concentration (mol m^−3^)
d: diameter (m)
D: diffusion coefficient (^m2^ s^−1^)
k: reaction coefficient (dep.)
kf: mass transfer coefficient (m s^−1^)
K: equilibrium coefficient (m^3^ mol^−1^)
m_cat_: catalyst mass ((k)g_cat_)
M: molar mass (g mol^−1^)
n: number of moles (mol)
n: reaction order (-)
r: reaction rate (mol (k)g_cat_^−1^ s^−1^)
t: time (s, h)
V: molar volume (cm^3^ mol^−1^)
V: reactor volume ((m)L)
x: mole fraction; lumped variable in Eq. (27) (mol mol^−1^; dep.)
X: conversion (mol mol^−1^)
y: defined in Eq. (27) (dep.)

**Greek symbols**
γ: activity coefficient (-)
ε: porosity (m^3^ m^−3^)
μ: viscosity (Pa s or cp)
π: lumped groups in Eqs. (25) and (26) (dep.)
ρ: density (kg m^−3^)
σ: error, confidence interval (dep.)
τ: tortuosity (m m^−1^)
φ: association parameter [5] (-)
Φ: Weisz modulus, see Eq. (5) (-)

**Subscripts**
0: initial
*: adsorbed
A: compound A
b: bulk
cat: catalyst
eff: effective
eq: equilibrium
i: compound i
p: particle
s: solute, surface
tot: total
w: volumetric basis

**Superscripts**
obs: observed

**Abbreviations and acronyms**
A: compound A, (oleic) acid
E: ester
M: methanol
W: water

Specifications table

**Table:** 

Subject	Chemical Engineering
Specific subject area	Catalysis Kinetic modelling of the heterogeneous esterification reaction of oleic acid into methyl oleate is performed on UiO-66 metal organic framework catalyst
Type of data	Graph Figure
How data were acquired	An amount of oleic acid (0.1 g, 0.139 g and 0.279 g) is dissolved in 1 mL methanol in a 1.5 mL GC vial, after which the UiO-66 catalyst is added at 10 mol% relative to the initial number of OA moles. The vial is closed and brought to reaction temperature (65, 75, 85°C) in a temperature controlled (± 0.1°C) oil bath (IKA, RET basic model) and in less than one minute the desired reaction temperature was reached. The stirring bar in the reaction vial is 7 mm long and has a 5 mm diameter. The stirring bar in the oil bath is 5 cm long and 1 cm wide. Stirrer speed was 600 rpm. Every hour, 8 μL is taken via a microsyringe (Hamilton 10 μL, made in Romania). The sample is filtered by a Jin Teng filter (PES, 13 mm/0.22 μm, made in China) and it is diluted in 0.5 mL hexane and 0.5 mL isopropanol in the sample vial. The reaction mixture is analyzed via injection of 1 μL, taken from the sample vial, via an Agilent 7890A GC with 25 μL of methyl heptadecanoate as an internal standard. The injection port is at 260°C, the pressure and total flow rate are 21.849 psi and 48.73 mL min^−1^ with a split ratio of 10:1. The GC detector temperature is 250°C. The repeatability of the given experimental protocol was checked. Blank experiments did not show significant OA conversion. The only product detected in the chromatogram for the catalytic experiments is methyl oleate.
Data format	Raw Analyzed
Parameters for data collection	The effects of reaction conditions were examined with 3 different initial oleic acid amounts (0.1 g, 0.139 g and 0.279 g) in 1 mL of methanol. Three temperatures levels were applied (65, 75 and 85°C). After model discrimination (based on 67 heterogeneous reaction rate expressions), using initial reaction rate analysis, intrinsic kinetic parameters were obtained by non-linear parameter estimation, using activity coefficients to account for the non-ideal behavior of the reaction mixture.
Description of data collection	Experimental gas chromatographic data for oleic acid are used to calculate the corresponding conversion, which serves as input for the initial reaction rate analysis and subsequent non-linear parameter estimation procedure.
Data source location	State Key Laboratory of Advanced Technology for Materials Synthesis and Processing, Wuhan University of Technology Wuhan China 30° 31′ 10.5′′ N, 114° 21′ 09′ 8′′ E
Data accessibility	With the article
Related research article	S. Chaemchuen, P.M. Heynderickx, F. Verpoort, Kinetic modeling of oleic acid esterification with UiO-66: from intrinsic experimental data to kinetics via elementary reaction steps, Chem. Eng. J. 394 (2020) 124816. https://doi.org/10.1016/j.cej.2020.124816 [1]

### Value of the data

• The presented data and corresponding data treatment can be put forward by other researchers in order to guarantee the acquisition of intrinsic experimental data for catalytic reactions.
• The presented data can be used as an example to set up typical heterogeneous esterification reactions by researchers working on catalytic systems with the specific purpose of kinetic modeling.
• Data treatment in order to calculate initial reaction rates is explained in detail with example. This has a high applicability and very easy practicability for users in the research field of heterogeneous catalysis.
• Concentration gradients, which might destroy the intrinsic character of the experimental data, can be ruled out via simple criteria. How to use the data is explained in this manuscript.

### 1 Data description

**Fig. 1:**
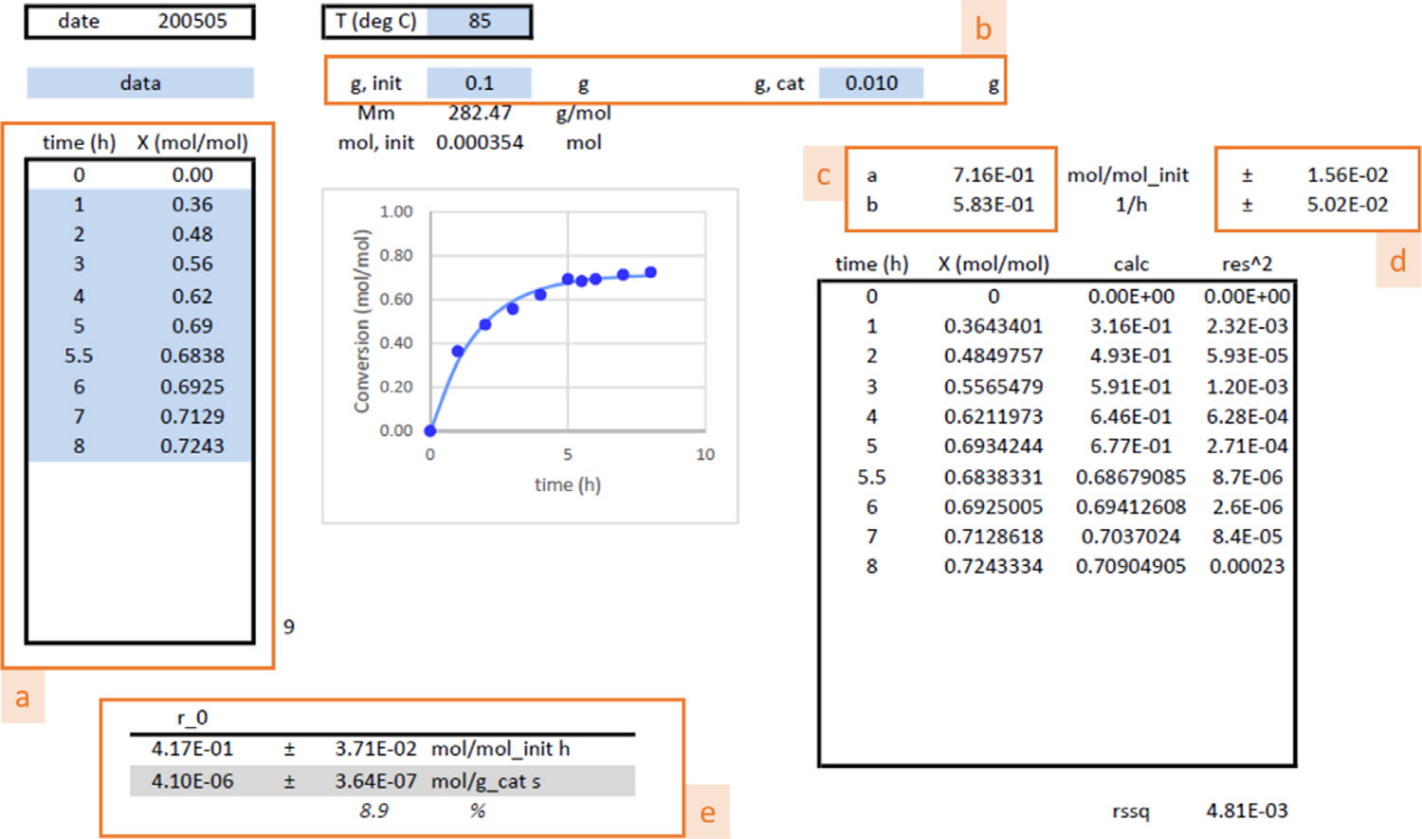
Calculation of the initial reaction rate with Eq. (2). Blue fields require input data. (a) conversion versus reactime data, (b) initial mass of catalyst and limiting reactant (oleic acid), (c) parameter estimates a and b in Eq. (1) after running Solver in Excel^Ⓡ^ (optimizing the rssq using calculations in black box), (d) parameter confidence intervals via the procedure, explained in de Levie [2] and (e) the value for te initial reaction rate, according Eq. (2). Specific details on these calculations can be found in the Supplementary Content of this paper.

**Fig. 2:**
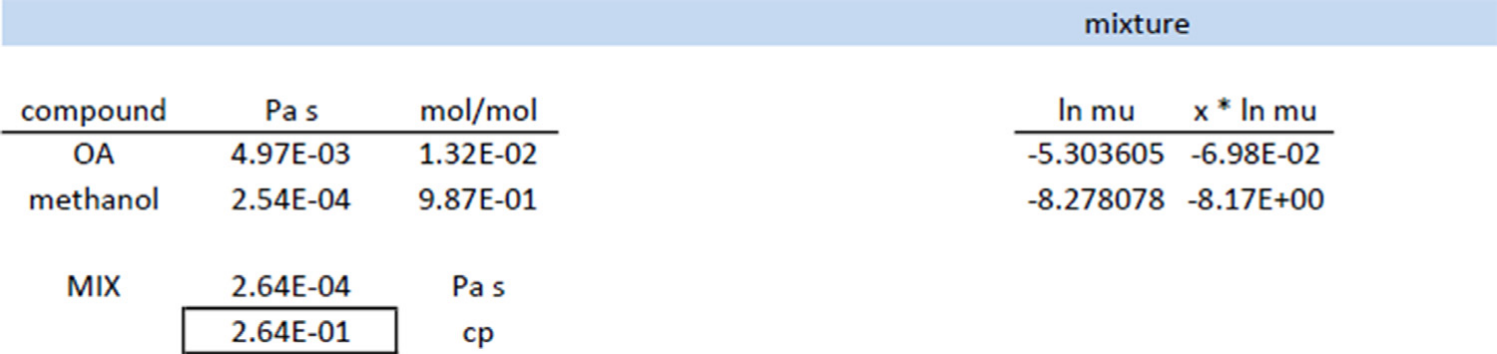
Calculation of viscosity of initial reaction mixture with Eq. (9).

**Fig. 3:**
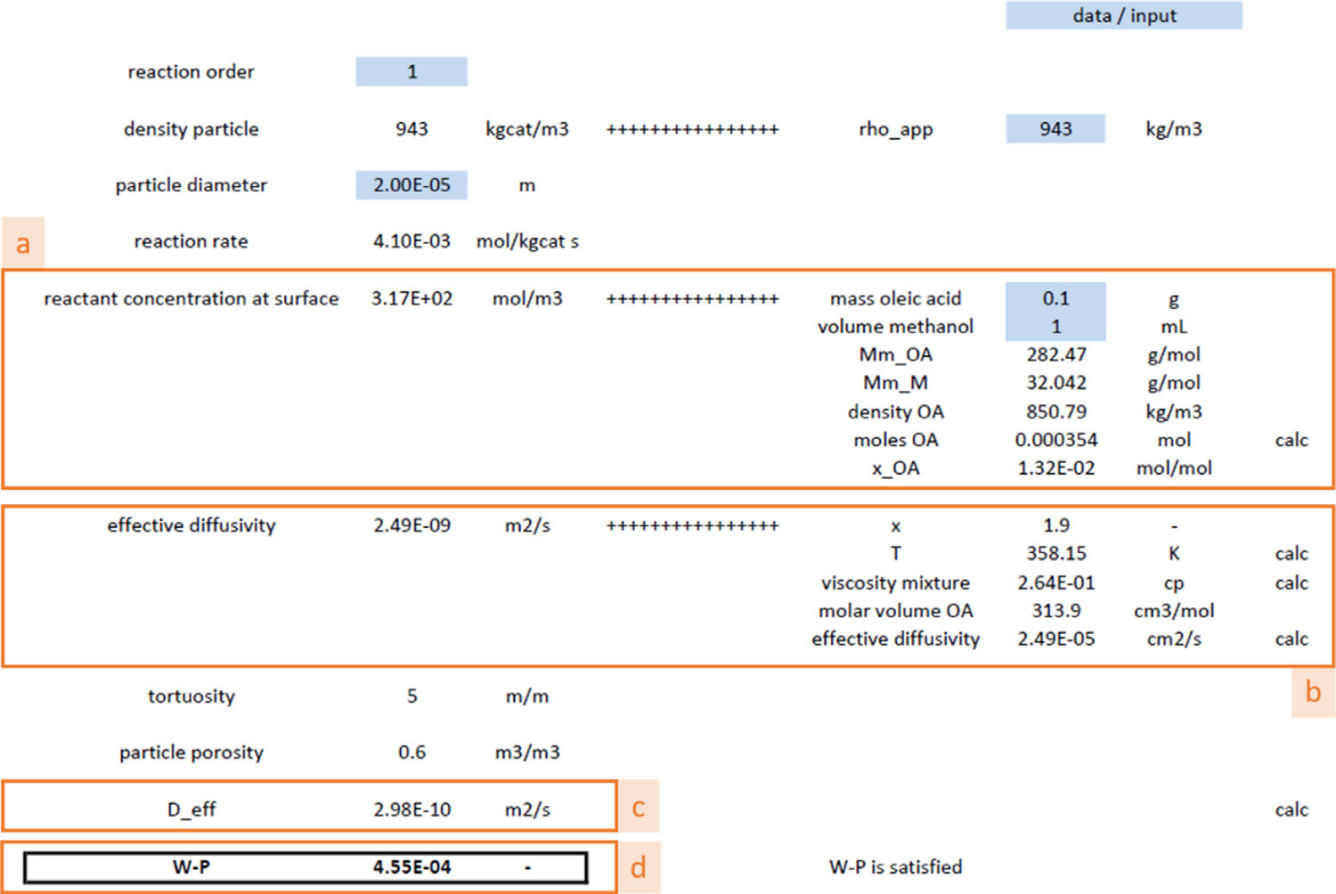
Evaluation of Weisz criterion with Eq. (5) (internal concentration criterion). Blue fields require input data and the viscosity value is taken from Fig. 2. (a) Surface concentration calculation, (b) calculation of effective diffusion coefficient, according to Eq. (7), (c) diffusion coefficient, according to Eq. (8) and (d) evaluation of the Weisz-Prater criterion, according to the left hand side of Eq. (5).

**Fig. 4:**
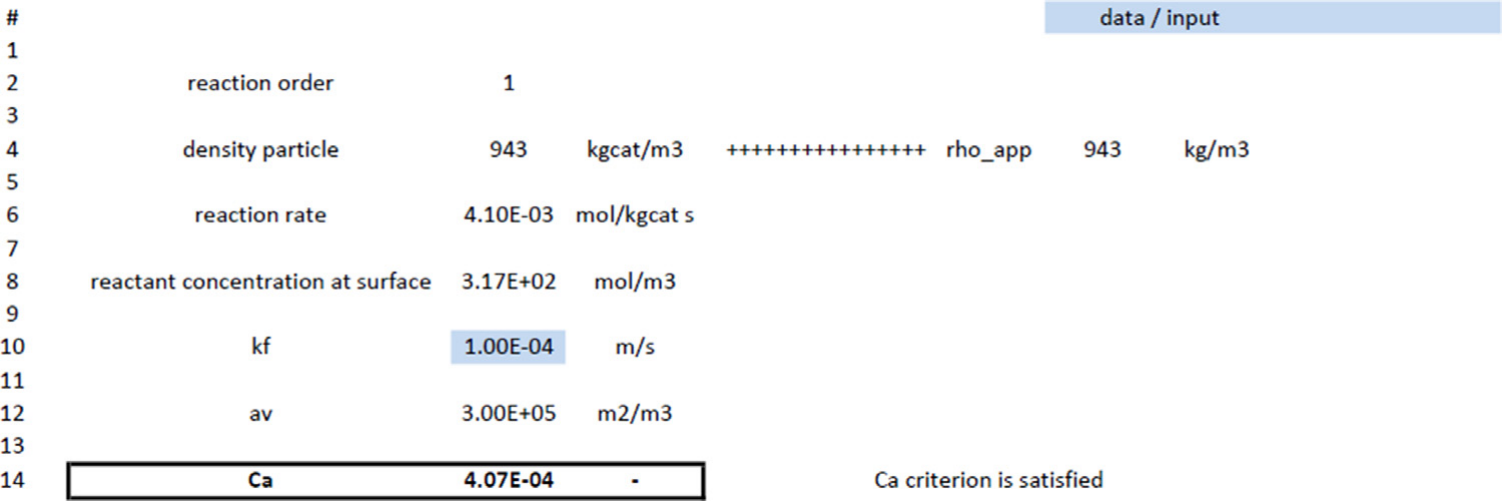
Evaluation of Carberry number with Eq. (6) (external concentration criterion). Blue fields require input data and other input is taken from Figs. 2 and 3.

This dataset contains 1 Table and 4 Figures in the main text. Table 1 contains the experimental conditions, together with the initial reaction rates and calculated Weisz modulus. Fig. 1 gives the details for the initial reaction rate calculation, corresponding to entry 6 in Table 1. Fig. 2 gives the details for the calculation of viscosity of the initial reaction mixture. The evaluation of Weisz criterion (internal concentration criterion) is given in Fig. 3, Fig. 4 provides the information for the evaluation of the Carberry number for the external concentration criterion.

**Table 1:** Experimental conditions for the esterification of oleic acid (OA) into methyl oleate (MO) using UiO-66 catalyst. V_M,__0_ = 1 mL, rpm = 600 min^−^^1^. *r_0_* and Φ are the initial reaction rate and the Weisz modulus, given by Eq. (5).

Entry	T (°C)	C_OA,0_ (M)	n_OA,0_ (mmol)	m_cat_ (mg)	r_0_ (μmol g_cat_^−^^1^ s^−^^1^)	Φ (10^−^^3^)
1	65	0.318	0.354	10.0	1.58 ± 0.05	0.230
2	65	0.426	0.492	13.9	1.56 ± 0.10	0.172
3	75	0.318	0.354	10.0	3.40 ± 0.25	0.426
4	75	0.426	0.492	13.9	2.03 ± 0.22	0.194
5	75	0.753	0.988	27.9	1.26 ± 0.21	0.072
6	85	0.318	0.354	10.0	4.10 ± 0.35	0.455
7	85	0.426	0.492	13.9	2.97 ± 0.27	0.251
8	85	0.753	0.988	27.9	1.94 ± 0.16	0.098

There are 7 additional Figures in Supplementary Content, analogous to Fig. 1, corresponding to the entries 1–5, 7 and 8 in Table 1.

### 2 Experimental design, materials and methods

First, this data paper explains how to obtain initial reaction rates from conversion versus reaction time data. Secondly, criteria for internal and external mass concentration gradients are given with explanation how to deal with them. Thirdly, details on heterogeneous catalytic rate expressions and corresponding initial reaction rate analysis from experimental data are given.

A reaction rate is maximal at zero conversion, i.e., the change in moles versus reaction time is highest at the beginning of the experiment. From an empirical relation between the conversion of the limiting reactant and time (in h), see Eq. (1), the initial reaction rate, r_0_ (in mol kg_cat_^−^^1^ s^−^^1^), is given by Eq. (2):

(1) $$X_{A}=a\cdot \left(1-\exp\left(-b\cdot t\right)\right)$$

(2) $$r_{A,0}=\frac{ab}{3600}\cdot \frac{n_{A,0}}{m_{cat}}$$

Parameters a and b are estimated via the Excel^Ⓡ^ Solver function, minimizing function S given by Eq. (3), and the confidence intervals are obtained via the procedure explained in de Levie [2].

(3) $$S\left(a,b\right)=\sum_{i}{\left(X_{A}-X_{A,calc}\right)}^{2}\;\to \;\min_{\left(a,b\right)}$$

The error on the reaction rate is obtained via Eq. (4):

(4) $$\sigma_{r_{A,0}}=r_{A,0}\cdot \sqrt{{\left(\sigma_{a}/a\right)}^{2}+{\left(\sigma_{b}/b\right)}^{2}}$$

On a side note, sometimes polynomial expressions are used to model the conversion versus reaction time data. In this case, the possibility exists that negative values for the first order term, corresponding to the initial reaction rate, are obtained. This is physically not acceptable and, therefore, expression (1) is preferred [3,4].

After the calculation of the initial reaction rate, the criteria for internal and external concentration gradients, given in Eqs. (5) and (6), can be evaluated [5]:

(5) $$\Phi =\left(\frac{n+1}{2}\right)\cdot \frac{r_{A,w}^{obs}\rho_{cat}}{D_{A,eff}C_{A,s}}\cdot {\left(\frac{d_{p}}{6}\right)}^{2}<0.08$$

(6) $$Ca=\frac{C_{A,b}-C_{A,s}}{C_{A,b}}=\frac{r_{A,w}^{obs}\rho_{cat}}{k_{f}a_{v}C_{A,b}}<\frac{0.05}{n}$$

It can be observed that Eqs. (5) and (6) directly rely on the value of the observed reaction rate, which has to be evaluated at its highest value in order to have proper validation. In Eqs. (5) and (6) the value for the observed reaction rate is expressed in mol m_cat_^−^^3^ s^−^^1^, so that correction for the density (ρ_cat_) is required.

Eq. (5) requires the input of the diffusion coefficient, see Eqs. (7) and (8); the former is the well-known Wilke and Chang correlation [6]:

(7) $$\frac{D_{A}\mu_{M}}{T}=\frac{7.4\cdot 10^{-8}{\left(\varphi_{S}M_{S}\right)}^{1/2}}{V_{A}^{0.6}}$$

(8) $$D_{A,eff}=D_{A}\cdot \frac{\varepsilon }{\tau }$$

The viscosity of the mixture (component A and solvent) is given by the Grunberg-Nissan mixing rule for liquid mixture [7], see Eq. (9):

(9) $$\ln\mu_{M}=\sum_{i=1}^{n}x_{i}\ln\mu_{i}$$

The molar volume of the limiting reactant, V_A_, can be found online or it can be estimated from group-contributive methods [8,9]. Effective diffusivities are then obtained with Eq. (8), with ε and τ the catalyst porosity and tortuosity.

Thus far, initial reaction rates are obtained and together with physical properties of the catalytic system, such as the viscosity of the solvent and the diffusion coefficient of the limiting reactant, the intrinsic character of the kinetic data are evaluated.

Lastly, the specific reaction rate expression is based on the Hougen-Watson formalism, using the Langmuir adsorption isotherm approach. The underlying assumptions may not always be completely fulfilled, but it is generally accepted that this approach is the most suitable and reliable way of rationalizing observed catalytic rate data [10,11].

The model for esterification reaction, as described in [1] considers the adsorption of oleic acid (A) and then methanol (M) reacts with the oleic acid adsorbate, see Eqs. (10) and (11). Surface reaction (11) addresses one additional active site to give the ester product (E) and water (W), both adsorbed on the catalyst surface, see Eqs. (12) and (13).

(10) $$A\;+\;*\;⇄\;A^{*}$$

(11) $$A^{*}\;+\;M\;+\;*\;⇄\;E^{*}\;+\;W^{*}$$

(12) $$E\;+\;*\;⇄\;E{}^{*}$$

(13) $$W\;+\;*\;⇄\;W^{*}$$

If the surface reaction (11) is rate determining, the overall reaction rate is given by Eq. (14) and relations (10), (12) and (13) provide the required equilibrium relations (15) to (17) to solve for the adsorbates:

(14) $$r=k_{s}C_{A^{*}}C_{M}C_{*}-k_{s}^{\prime }C_{E^{*}}C_{W^{*}}$$

(15) $$K_{1}=\frac{C_{A^{*}}}{C_{A}C_{*}}$$

(16) $$K_{2}=\frac{C_{E^{*}}}{C_{E}C_{*}}$$

(17) $$K_{3}=\frac{C_{W^{*}}}{C_{W}C_{*}}$$

Together with the active site balance, see Eq. (18), the esterification reaction rate expression, Eq. (19), can be established, taking into account non-ideality of the liquid phase, where concentration (C) is replaced by activity (a):

(18) $$C_{*}+C_{A^{*}}+C_{E^{*}}+C_{W^{*}}=C_{tot}$$

(19) $$r=\frac{k_{s}K_{1}C_{tot}^{2}}{{\left(1+K_{1}a_{A}+K_{2}a_{E}+K_{3}a_{W}\right)}^{2}}\cdot \left(a_{A}a_{M}-\frac{a_{E}a_{W}}{K_{eq}}\right)$$

Activity coefficients, γ, link concentration to the compounds’ activity, via a = γ∙C. They are calculated based on the composition of the reaction mixture [12].

Oleic acid is the limiting reagent, so the concentrations (to be converted into activities) can be written as Eqs. (20) to (22):

(20) $$C_{A}=C_{A,0}\cdot \left(1-X\right)$$

(21) $$C_{M}=C_{M,0}-C_{A,0}\cdot X$$

(22) $$C_{E}=C_{W}=C_{A,0}\cdot X$$

The initial rate is evaluated at zero conversion, X = 0, so that Eq. (19) is replaced by Eq. (23):

(23) $$r_{0}=\frac{k_{5}K_{1}C_{tot}^{2}}{{\left(1+K_{1}a_{A,0}\right)}^{2}}\cdot a_{A,0}a_{M,0}$$

Eq. (23) can be linearized by taking the square root of both sides and subsequent inversion, and after rearranging terms, Eq. (24) is obtained with substitutions (25) and (26):

(24) $$\frac{C_{tot}}{\sqrt{\;r_{0}}}\cdot \sqrt{\;a_{A,0}a_{M,0}}=\pi_{1}+\pi_{2}\cdot a_{A,0}$$

(25) $$\pi_{1}=\frac{1}{\sqrt{\;k_{5}K_{1}}}$$

(26) $$\pi_{2}=\sqrt{\;\frac{K_{1}}{k_{5}}}$$

All Hougen-Watson expressions can be transformed into a linear function as presented in Eqs. (23) and (24). The latter is a linear relation and the initial data can be evaluated very quickly in Excel^Ⓡ^ via the ‘linest’ function. This function deals with linear regression models such as given in Eq. (27):

(27) $$y=b_{0}+\sum_{i=1}^{n}b_{i}x_{i}$$

The parameters b, representative for the lumped parameters π in Eq. (24), should be positive and significant in order to generate valid models able to describe the data set. If a parameter bj (j = 0…n) is not significantly different from 0, it must be set to zero. In this case, the considered model can describe the whole data set, but in the original model the corresponding parameter should be set to zero. In the case that a parameter bj is significantly negative, this model will not make physical sense (kinetic parameters cannot have negative value) and it should be discarded from the list for the current data. Hence, the given transformation procedure can be successfully used for model discrimination, as described in previous reports [4,10].

It can be added that the correct input of the initial reaction rate, as determined via Eq. (2) from raw experimental conversion versus reaction time data, is required to evaluate the initial reaction rate analysis as a discrimination tool via Eq. (24).

### 3 Data and results

For the calculation of the initial reaction rate, Eq. (2) is used based on the conversion versus reaction time data fitting with Eq. (1), as shown in Fig. 1. This calculation requires only the data (reaction time, conversion), together with initial mass of limiting reactant and the catalyst mass. Note that the variance for coefficients A and B, taken as the confidence interval after estimation, is obtained via an in-house built protocol, following reference [2], because Excel^Ⓡ^ only has built-in parameter estimation without providing the corresponding confidence intervals.

The mass of the limiting reactant (oleic acid) and the volume of solvent (methanol) is taken as input for the calculation of the mixture viscosity, see Fig. 2, at the given temperature. If required, temperature dependency per compound should be done in advance.

Fig. 3 shows the evaluation of Weisz criterion to rule out internal concentration criterion via Eq. (5). The effective diffusivity is calculated using Eqs. (7) and (8).

Fig. 4 shows the evaluation for the possible absence of external concentration gradient according to the Carberry number calculation. Note that this criterion needs to be satisfied in order to calculate the surface concentration based on bulk properties, see Fig. 3.

## Declaration of Competing Interest

The authors declare that they have no known competing financial interests or personal relationships which have, or could be perceived to have, influenced the work reported in this article.

## Appendix Supplementary materials

Image, application 1

Image, application 2
